# Early-life conditions and health at older ages: The mediating role of educational attainment, family and employment trajectories

**DOI:** 10.1371/journal.pone.0195320

**Published:** 2018-04-05

**Authors:** Bruno Arpino, Jordi Gumà, Albert Julià

**Affiliations:** Department of Political and Social Sciences, Universitat Pompeu Fabra, Barcelona, Spain; University of Kentucky, UNITED STATES

## Abstract

**Objectives:**

We examine to what extent the effect of early-life conditions (health and socioeconomic status) on health in later life is mediated by educational attainment and life-course trajectories (fertility, partnership, employment).

**Methods:**

Using data from the Survey of Health, Ageing and Retirement in Europe (*N* = 12,034), we apply, separately by gender, multichannel sequence analysis and cluster analysis to obtain groups of similar family and employment histories. The KHB method is used to disentangle direct and indirect effects of early-life conditions on health.

**Results:**

Early-life-conditions indirectly impact on health in later life as result of their influence on education and family and employment trajectories. For example, between 22% and 42% of the effect of low parental socio-economic status at childhood on the three considered health outcomes at older age is explained by educational attainment for women. Even higher percentages are found for men (35% - 57%). On the contrary, the positive effect of poor health at childhood on poor health at older ages is not significantly mediated by education and life-course trajectories. Education captures most of the mediating effect of parental socio-economic status. More specifically, between 66% and 75% of the indirect effect of low parental socio-economic status at childhood on the three considered health outcomes at older age is explained by educational attainment for women. Again, higher percentages are found for men (86% - 93%). Early-life conditions, especially socioeconomic status, influence family and employment trajectories indirectly through their impact on education. We also find a persistent direct impact of early-life conditions on health at older ages.

**Conclusions:**

Our findings demonstrate that early-life experiences influence education and life-course trajectories and health in later life, suggesting that public investments in children are expected to produce long lasting effects on people’s lives throughout the different phases of their life-course.

## Introduction

A growing literature has pointed at long lasting influences of early-life conditions on individuals’ health and survival. Exposures to diseases or to a context of deprivation in terms of low socioeconomic conditions have been found to have enduring consequences on health [1–4]. Another strand of the literature suggests a substantive influence of family [5–7] or employment [8] trajectories on health, wellbeing and mortality. In addition, other studies have uncovered important effects of early-life conditions on partnership formation and dissolution [9], human reproduction [10] and employment trajectories [11]. These studies highlight that individuals are likely to be selected in their family and employment trajectories based on the conditions they experienced during childhood in terms of health and socioeconomic status (SES).

In this study we bring together these different strands of the literature. Our goal is to examine to what extent early-life influences are mediated by subsequent life-course experiences, and in particular, by educational attainment and family and work histories. We also aim at examining the effect of early-life conditions on life-course trajectories and whether this is mediated by educational attainment. We adopt a holistic approach to the life course by considering early-life course conditions and employing multichannel sequence analysis to examine different aspects of individuals’ trajectories during adulthood (quantum, timing, sequence) and different dimensions (fertility, partnerships, employment) simultaneously.

## Background

### Early-life conditions

Research suggests that conditions during early-life and childhood are associated with subsequent life stages and life course [12]. Previous studies showed that family SES [2, 3] and health during childhood have long-term effects on health during adulthood [13].

Early-life conditions may influence health at older ages *via* different mechanisms [14]. According to the Critical Period Model [15] early-life circumstances may exert long-lasting effects on health via a biological imprinting mechanism, affecting adult health independently of intervening experiences. The pathway model suggests, instead, that early-life environment shapes subsequent life-course experiences that in turn affect health status at older ages [16]. Early-life conditions may also interact with individuals’ achievements at later life-course stages (Accumulation of Risks and the Social Mobility models; [4]).

Growing in a family with low SES (poor economic resources and/or low educational levels), has displayed a noteworthy association with lower probabilities of achieving a university degree and higher chances of discontinuous employment trajectories [17, 18]. Recent studies also reported that early-life conditions are associated with subsequent family patterns at adult ages. Van den Berg and Gupta [9] found that the socioeconomic context around ages 7–12 was associated with marriage rates of men but not of women. Sloboda et al. [10] found that exposure to poor nutritional context during childhood affected the process of reproductive evolution (age at menarche, fertility capacity, menopause, etc.) of women, impacting on their fertility.

Finally, the role of health as a factor which influences individuals’ probabilities of finding a partner has been largely explored. Those with a poorer health profile show lower chances of starting a union and higher probabilities of breaking up a union during their life-course than their counterparts with better health status [19].

In summary, early-life conditions may influence educational attainment and life-course trajectories during adulthood which have shown to be associated with health in later life. Therefore, it is of interest to examine the effect of early-life conditions that can be explained through their influence on educational attainment and life-course trajectories (fertility, partnership, employment).

### Education as a mediator of early-life influences

Early-life conditions may influence educational attainment that in turn influences life-course trajectories and thus health and wellbeing [1, 4, 20]. Caro et al. [18], for example, found that family SES positively influences educational attainment and occupational status of children. Previous studies reported a positive association between educational attainment and delays in marital and fertility trajectories for both sexes,. Education has been also found to be positively associated with cohabitation [21] and childlessness, but only for women [22]. In addition, education has also showed gender differences in the magnitude of its association with health, being this higher among women [23].

### Family histories and health

The bulk of the literature on family histories and health has focused on a single aspect, such as, partnership [24, 25] and fertility [5, 6].

More recent research has considered the complexity of family histories adopting a holistic perspective (with respect to timing, quantum, ordering) when analyzing health in different stages of individual’s life course. O’Flaherty et al. [26], for example, explored the link between physical health at older ages and a comprehensive reconstruction of family trajectories based on both fertility and partnership histories from age 18 to 50. They found that men’s health at older ages seems to be more sensitive to uncommon family trajectories (early family formation, long life singlehood, early marital disruption, or high fertility) than women’s. Among women, only those who experienced both a disrupted marital history and a high level of fertility were found to show considerably poorer health than other groups of women.

### Work histories and health

Unemployment has been found to negatively influence in the medium and long term physical [27] and mental health [28]. Instead, the effect of retirement on health depends on which was the individual’s status before retirement. Retiring from employment is generally associated with health deteriorations [29], whereas when the initial status was unemployment or inactivity the effect of retirement seems to be null or even positive [30].

The magnitude of the effect of employment trajectories on health has proved to be dependent on gender, being stronger among men due to the central role that employment plays for male identity [31].

### Research questions

**Fig 1:**
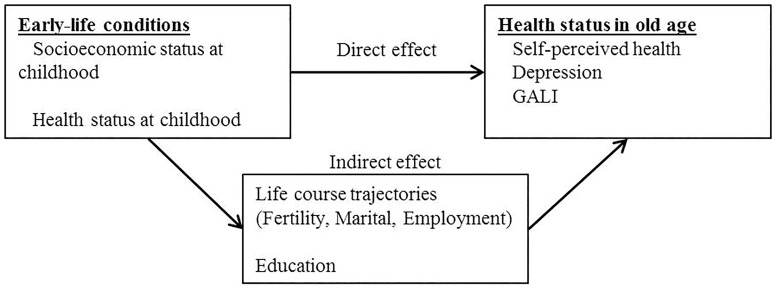
The direct and indirect effect of early-life conditions on later-life health.

**Fig 2:**
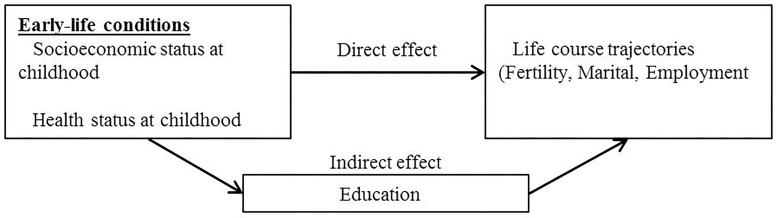
The direct and indirect effect of early-life conditions on life-course trajectories.

Although previous studies have illuminated on different aspects of the life course influences on later-life health, there are still gaps to be filled. Most importantly, the literature on the long-term effect of early-life conditions has overlooked the mediating effect of subsequent family and employment life trajectories, while the limited literature on the effect of life course trajectories on health at older ages has ignored the role of early-life course conditions. Putting together the different strands of the literature summarized above, we argue that early-life conditions may have a domino effect influencing individuals’ probabilities of attaining a certain educational level, which influences subsequent employment and family trajectories [17, 18], which may impact on health at older ages. Our conceptual scheme is illustrated in Figs 1 and 2 that also illustrate the two main parts of our empirical analyses.

Fig 1 shows that we are interested in estimating what part of the effect of early-life conditions on health is mediated by educational attainment and life-course trajectories. In a second step (Fig 2), we deepen the analyses of the mediating role of educational attainment and examine to what extent the impact of early-life conditions on life-course trajectories is mediated by educational attainment. In our conceptual scheme, thus, educational attainment has a double role as mediator: in between early-life conditions and health status at older ages; and in between early-life conditions and life-course trajectories. We implement all the analyses separately by gender because the literature summarized above suggests important gender differences in all considered relationships.

## Materials and methods

### Data

We use data from the first three waves of the Survey of Health, Ageing and Retirement in Europe (SHARE). SHARE has been reviewed and approved by the Ethics Committee of the University of Mannheim [32] and all participants provided written consent. SHARE is a panel survey representative of the non-institutionalized population aged 50 and over carried out in different European countries [33]. Registered users can download the data for free from the website http://www.share-project.org/. All data provided by SHARE are fully anonymized. More info on data use and confidentiality can be found at: http://www.share-project.org/data-access/share-conditions-of-use.html.

The third wave of SHARE (2008/09), called SHARELIFE, only collected detailed retrospective information (i.e., on fertility histories and early-life conditions). From the third wave we obtained information on early-life conditions and life course trajectories. Outcomes and other variables were measured in the second wave of SHARE (2006/07). In case of missing data on these variables we used information from the first wave (2004).

We restricted our sample to individuals aged at least 60 years (which correspond to the average effective age at retirement) and reconstructed complete employment and family trajectories between ages 15 and 59 (both included). As a robustness check we also run the analyses using 65 as the age cut-off but results were very similar to those presented below. We dropped individuals born abroad and few cases of homosexual couples. Our working sample is composed by 12,034 individuals (6,221 women; 5,813 men).

### Variables

#### Early-life conditions

We considered two dimensions of early-life conditions: health and SES. Health during childhood (defined as from when respondents were born up to age 15) was measured in SHARELIFE asking: “Would you say that your health during your childhood was in general: excellent, very good, good, fair, poor or health varied a great deal”. We dichotomized this variable distinguishing between those who declared poor health (= 1; fair, poor, health varied a great deal) or good health (= 0, otherwise) during childhood.

SES was measured using several items that refer to the situation when the respondent was aged 10: overcrowding rate of the household (i.e., number of household members over the number of rooms); occupation of main breadwinner, treated as an ordinal variable; number of books in the household. We summarized these variables using a principal component analyses that returned a single factor which accounted for the 51% of the whole variability. We chose to work with only one factor for the sake of simplicity as well as because this was the only factor which presented an eigenvalue higher than 1 (the eigenvalues for the second and third factor were 0.87 and 0.61 respectively). The final factor was divided into four quartiles (“high SES”; “medium-high SES”, “medium-low SES”; “low SES”)

All early-life conditions variables that we use can be affected by recall bias. However, Havari and Mazzonna [34] assessed the internal and external consistency of the SHARELIFE measures of childhood health and socio-economic status we use and found that overall respondents seem to remember fairly well their childhood conditions.

#### Family and employment trajectories

We measured three dimensions of individuals’ life-course trajectories: *Fertility*; *Marital status* and *Employment* every year between ages 15 and 59 (see below for details).

#### Education

We considered three educational groups based on the level of education achieved using the International Standard Classification of Education (ISCED): “low” (corresponding to ISCED 0–2, lower secondary education or lower; reference), “medium” (ISCED 3–4, higher secondary education) and “high” (ISCED 5–6, tertiary education).

#### Dependent variables

**Self-perceived health**: This is a holistic measure of individuals’ general health status included in SHARE questionnaire with five possible answers: "Excellent", "Very good"; "Good"; "Fair"; "Poor”. Following the common practice in the literature [35], we transformed this variable into a binary variable indicating whether self-perceived health is “bad” (= 1, corresponding to "fair" or "poor") or “good” (= 0, otherwise).

**European depression scale (Euro-D)**: This variable was selected to measure respondents’ mental health. It measures the number of depressive symptoms reported by the respondent and ranges from 0 to 12.

**GALI**: The Global Activity Limitation Indicator (GALI) belongs to the family of disability indicators, targeting situations in which health disorders and conditions have impacted people’s usual activities [36]. Information comes from the question: “For the past six months at least, to what extent have you been limited because of a health problem in activities people usually do? Severely limited; Limited, but not severely; Not limited “. We grouped these categories to indicate whether a person is somewhat limited (= 1) or not (= 0).

#### Control variables

In all regression models we control for *country of residence* in order to account for country differences and *age* at the time of the survey categorized in 5-year ranges (60–64—reference category; 65–69; 70–74; 75–79; 80+). Controlling for age as a continuous variable did not make any difference. Model fit remained virtually unchanged when using age as categorical variable as compared to models where age was used as continuous, for all health outcomes and both genders. The ratio of the R-square (or pseudo R-square) for the two types of models was about 1, with the only exception of the multinomial regression for women where the ratio of the pseudo R-squares was 0.9 (lower explanatory power for the model with age as a continuous variable).

### Analytic strategy

Our empirical approach consists of two steps. First, by applying sequence and cluster analyses, we obtain clusters of similar life-course trajectories separately by gender. Second, we assess the effect of early-life conditions on health at later life and the mediating role of education and life-course trajectories.

#### Sequence and cluster analysis

We adopt Sequence Analysis (SA; [37]) to describe life-course trajectories between ages 15 and 59. SA defines life-courses as sequences of events allowing to simultaneously account for timing, quantum and ordering of individuals’ experiences. We use multichannel sequences analysis (MCSA; [38]) to be able to represent the interconnectedness of life events in relation to three dimensions: fertility, partnerships and employment. More specifically, each year between ages 15 and 59 (both included) we measure: *Fertility* (0, 1, 2, and 3 or more children); *Marital status* (married, cohabitating, divorced/separated, widowed, single); and *Employment* (in education, employed full-time, employed part-time, unemployed, retired, other inactive). The term “inactive” only refers to the labour market and it does not exclude that the individual is active in other spheres of life.

Once all individuals’ trajectories were defined for each of the three dimensions, we calculated (dis)similarities among them using a dynamic algorithm known as Optimal Matching Analysis (OMA; [39]). The similarity between two sequences is determined by calculating the total “costs” of turning one sequence into another based on three operations: insertion, deletion, and substitution. Following previous studies (e.g., [40]), insertion and deletion costs are set to one and substitution costs are empirically defined as the inverse of the transition rates, so that the more common the transition between two states observed in the data, the lower the substitution cost. This operation provides three distance matrixes (one per dimension). MCSA calculates the overall distance matrix as the average of these three matrixes [38].

Next, we applied hierarchical Cluster Analysis (CA) using Ward ’s minimum variance criterion to the distance matrix to obtain clusters of trajectories [41]. The goal of CA is to identify a limited number of groups of life-course trajectories that can be interpreted and analyzed in a meaningful way [40]. More specifically, we aimed at isolating clusters of individuals whose family and employment trajectories were the most similar possible within the clusters and dissimilar compared to individuals’ trajectories in other clusters. MCSA, OMA and CA were implemented, separately by gender, using the R package *TraMineR* [42].

An important step in cluster analysis is to determine the number of clusters. A completely satisfactory and universally accepted solution to determine the number of clusters is not available in the literature [43]. Following common practice in cluster analysis, in order to choose the number of clusters we combined insights from statistical fit measures (specifically, the average silhouette width, ASW; [41]), and from the visual inspection of the dendrogram, with the need of obtaining a substantively interpretable solution. We, therefore, opted for a 12-cluster solution for women and a 6-cluster solution for men, which corresponded to the highest ASW for women (ASW = 0.2) and men (ASW = 0.47) across the different cluster solutions, respectively. Having 12 clusters for women implies reduced sample sizes in some clusters. As a robustness check, we considered a more parsimonious cluster solution for women (6 clusters) that however did not alter appreciably our conclusions. The obtained cluster solutions resulted also in two sets of interpretable clusters from a substantive point of view.

#### Mediation analysis of the effect of early-life conditions

To quantify what part of the effect of early-life conditions on health can be attributed to educational attainment and clusters of life-course trajectories (identified in the previous step), we used the “KHB” method (the acronym refers to the surnames of the three authors; [44]). This method appropriately compares estimates from a regression model where health outcomes are regressed on early-life conditions and control variables but not the mediators (*unadjusted*) with the estimates from the same model that adjusts also for education and life-course trajectories (*adjusted*). In this way, the KHB method allows calculating what part of the effect of early-life conditions on health in later-life is mediated by education and life-course trajectories (*indirect effect*; see Fig 1). The KHB method also allows using multiple mediators and decomposing the indirect effect due to all mediators in the part due to each of them separately, i.e. different educational levels and clusters of life-course trajectories. The effect of early-life conditions that is left after adjusting for the mediators is defined as *direct effect* and corresponds to the estimates of the adjusted model. The *total* effect is the sum of the direct and indirect effects and corresponds to the regression estimates in the unadjusted model.

Two of our health outcomes are binary (self-perceived health and GALI) while the depression scale is numerical. Therefore, we estimated logistic regression models for the two former and a linear regression model for depression. All models were estimated separately for women and men.

In the last step of our analyses, we aimed at deepening our understanding of the mediating role of educational attainment and we apply the “KHB” method to examine whether early-life conditions impact on life-course trajectories through educational attainment (Fig 2). In this case we estimated (unadjusted and adjusted) multinomial regression models to assess the effect of early-life conditions on the probability of having experienced a certain type of life-course during adulthood (represented by the clusters of trajectories) and the mediating role of education. For technical details about the KHB method, we refer the reader to Karlson, Holm and Breen [44] and Breen, Karlson and Holm [45] which cover the theory behind the method, the formulas for decomposing total, direct and indirect effects and the discussion about comparisons with other methods. We implemented the KHB method in Stata 14 using the *khb* package [46].

## Results

### Sequence and cluster analysis

Summary statistics on life-course trajectories for each cluster are presented in S1 Table, and their graphical representation in S1 and S2 Figs. The description of the clusters reveals a great variety of family and employment trajectories for both women and men. In the following we summarize the main features of each cluster.

Among women, the cluster “*No union*, *inactive”* is characterized by a high prevalence of women that never lived with a partner (88.3%) and by a significant proportion of women that have never worked (43.3%). Many women in this cluster did not have children (39.2%).

The cluster “*Children 3+*, *inactive”* is characterized by a traditional profile: a relatively early entry into first partnership (mean age 24.3), an average of 3.9 children and a high proportion of not employment (44.9%). The clusters “T*wo children*, *inactive”* and *“One child*, *inactive”* show very similar characteristics but with a lower average number of children (2.0 and 1.0, respectively). They are also characterized by higher age at entry into first union (25.6 and 27.7, respectively).

Women in the clusters “*Children 3+*, *married”* and “*Children 3+*, *part-time”* hold the lowest mean age at entry into the first partnership (23.4 and 23.9) and high fertility (3.7 and 3.4 average number of children, respectively). Both clusters are characterized by relatively high proportions of years spent in employment (on average 31.5 and 29.3), but women in the first cluster worked mostly full-time, while their counterparts in the other cluster worked frequently part-time. The cluster “Two *children*, *part-time”* is analogous to “*Children 3+*, *part-time”*, but with a lower fertility (2.0). Similarly, the clusters “*Two children*, *married” and* “*One child*, *married”* are characterized by a lower average number of children (2.0 and 1.0, respectively).

The clusters “*No children*, *married*, *employed*” and “*No children*, *married*, *inactive*” are composed by childless women who started living with a partner later than women in other clusters. They diverge markedly with respect to employment: whereas all women in the first cluster have worked, 41.8% of women in the second one never worked.

Finally, women in the cluster “*No children*, *no union*” are mostly childless women who never lived with a partner and spent, on average, the highest amount of years in employment (36.1).

As it is well known, men experience partnership formation and fertility events later than women (by three years on average). They also report a considerably lower prevalence of inactivity at age 59 as compared to women (36.2% vs 68.2%). The number of clusters for men is much lower because they show a considerably higher degree of homogeneity in their employment trajectories than women. Only a small cluster of men (about 2% of all men; “*Low employment*”) displayed a sizeable percentage of individuals who never worked (48.1%). This is also the only cluster of men where part-time employment is not negligible.

The other clusters differ with respect to timing and quantum of fertility and partnership. The clusters “*Children 3+*, *married”*, “*Two children*, *married”* and “*One child*, *married”* differ essentially in their fertility (average number of children is 3.6, 2.0 and 1.0, respectively). All men in these clusters have experienced living with a partner, however *“One child*, *married”* men started their first union relatively late (mean age 26.9) and had their first child late (mean age 30.0).

The remaining clusters, “*No children*, *no union*” and “*No children*, *married*” are almost entirely composed by childless men. They diverge with regard to partnership histories: 82.2% of men in the “*No children*, *no union*” cluster never lived with a partner, while this percentage drastically reduces to 6.2% for the “*No children*, *married*” cluster. Moreover, if men in the “*No children*, *no union*” cluster formed a union, this was virtually in every case a cohabitation.

### Mediation analysis of the effect of early-life conditions

Tables 1 and 2 present the results for women and men, respectively, of the first part of the empirical analyses corresponding to Fig 1. These tables report the results from the application of the KHB method to estimate the direct and indirect effect of early-life conditions on three health measures. The proportions of direct and indirect effects obtained from these estimates are reported in S2 Table. In these regression analyses, the clusters of life-course trajectories were treated as categorical variables. In other words, we have considered a set of 11 dummy variables for women and of 5 dummy variables for men and considered the clusters “*No union*, *inactive*” and “*Children 3+*” as reference category in the models for women and men, respectively.

**Table 1:** Estimated linear and logistic regression coefficients (standard errors in parentheses of SES and Health at 10 (early-life conditions) on three measures of health and mediating effect of life course trajectories and education. Women. (N = 6,221).

Outcomes:	Self-perceived health				Depression				GALI			
Early-life conditions:	Medium-high SES	Medium-low SES	Low SES	Poor Health	Medium-high SES	Medium-low SES	Low SES	Poor Health	Medium-high SES	Medium-low SES	Low SES	Poor Health
Total effect	0.272**	0.513***	0.677***	1.047***	0.090	0.332***	0.622***	0.843***	0.067	0.220**	0.419***	0.711***
	(0.079)	(0.080)	(0.086)	(0.101)	(0.079)	(0.081)	(0.088)	(0.099)	(0.076)	(0.078)	(0.084)	(0.096)
Direct effect	0.158†	0.359***	0.504***	1.034***	0.004	0.204*	0.483***	0.825***	-0.040	0.069	0.247**	0.701***
	(0.081)	(0.083)	(0.090)	(0.101)	(0.081)	(0.085)	(0.092)	(0.099)	(0.078)	(0.081)	(0.088)	(0.097)
Indirect effect	0.114	0.153*	0.173*	0.012	0.086	0.128*	0.139*	0.018	0.107†	0.151*	0.172**	0.010
	(0.071)	(0.073)	(0.074)	(0.069)	(0.060)	(0.063)	(0.064)	(0.058)	(0.063)	(0.065)	(0.066)	(0.061)
*Clusters*	Contribution				Contribution				Contribution			
Children 3+, inactive		2.8	4.0			-1.6	-2.5		0.0	0.1	0.1	
Children 3+, married		1.7	5.7			-0.4	-1.4		0.4	1.4	4.7	
Two children, married		-1.3	-2.5			2.2	4.2		-0.8	-1.0	-1.8	
One child, married		-2.5	-0.7			1.2	0.4		-0.6	-0.9	-0.3	
Children, no union		-5.2	-4.4			1.9	1.7		0.0	0.0	0.0	
No children, mar., employed		-3.5	-2.8			-1.9	-1.6		-0.5	-1.5	-1.2	
One child, inactive		-1.2	-0.9			-0.1	-0.1		0.4	-0.3	-0.2	
Two children, inactive		7.6	0.5			0.2	0.0		3.3	3.6	0.2	
Children 3+, part-time		-0.2	0.3			2.0	-2.3		0.5	-0.4	0.5	
No children, married, inactive		1.1	1.4			-0.1	-0.1		0.1	-0.4	-0.5	
Two children, part-time		-0.6	0.0			0.1	0.0		-0.2	0.1	0.0	
*Education*												
Medium		19.6	24.5			27.7	36.1		14.8	25.6	31.7	
High		81.8	74.9			68.9	65.6		82.6	73.6	66.6	

*Note*. All models control for age and country. A logistic regression model was estimated for self-perceived health and GALI, while a linear regression model was employed for depression. Reference categories for SES and Health are “High SES” and “Good Health”, respectively. Contributions, i.e., the portions of the indirect effect attributable to each mediators (life course trajectory clusters and education) are only displayed when the indirect effect was significant.

*** p<0.001;

** p<0.01;

* p<0.05;

^†^ p<0.1.

Inactive refers to the labour market only.

**Table 2:** Estimated linear and logistic regression coefficients (standard errors in parentheses) of SES and Health at 10 (early-life conditions) on three measures of health and mediating effect of life course trajectories and education. Men. (N = 5,813).

Outcomes:	Self-perceived health				Depression				GALI			
Early-life conditions:	Medium-high SES	Medium-low SES	Low SES	Poor Health	Medium-high SES	Medium-low SES	Low SES	Poor Health	Medium-high SES	Medium-low SES	Low SES	Poor Health
Total effect	0.30***	0.47***	0.49***	0.72***	0.11†	0.22**	0.29***	0.50***	0.05	0.23**	0.27**	0.50***
	(0.08)	(0.08)	(0.09)	(0.11)	(0.07)	(0.07)	(0.08)	(0.10)	(0.08)	(0.08)	(0.09)	(0.11)
Direct effect	0.19*	0.33***	0.32**	0.71***	0.07	0.14†	0.19*	0.49***	-0.04	0.11	0.12	0.50***
	(0.09)	(0.09)	(0.09)	(0.11)	(0.07)	(0.07)	(0.08)	(0.10)	(0.08)	(0.08)	(0.09)	(0.11)
Indirect effect	0.11	0.15*	0.18*	0.01	0.05	0.08†	0.10*	0.01	0.08	0.12†	0.16*	0.00
	(0.07)	(0.07)	(0.07)	(0.07)	(0.04)	(0.04)	(0.05)	(0.04)	(0.06)	(0.06)	(0.07)	(0.06)
*Clusters*	Contribution				Contribution				Contribution			
No children, no union		0.6	1.7			0.7	1.7			-0.1	-0.3	
No children, married		-0.1	-0.4			0.2	0.7			0.2	0.8	
One child, married		-1.5	-1.7			-0.7	-0.7			0.7	0.8	
Two children, married		-0.1	-0.3			0.6	2.7			0.5	2.3	
Low employment		0.4	0.0			0.5	0.0			-0.2	0.0	
*Education*												
Medium		2.5	8.1			2.7	8.3			3.5	10.8	
High		98.1	92.6			96.1	87.3			95.4	85.7	

Note: All models control for age and country. A logistic regression model was estimated for self-perceived health and GALI, while a linear regression model was employed for depression. Reference categories for SES and Health are “High SES” and “Good Health”, respectively. Contributions, i.e., the portions of the indirect effect attributable to each mediators (life course trajectory clusters and education) are only displayed when the indirect effect was significant.

*** p<0.001;

** p<0.01;

* p<0.05;

^†^ p<0.1.

Tables 1 and 2 are organized in three different vertical block, one for each of the three considered outcome variables. Within each block, the first part presents the total, direct and indirect effects of each early-life condition variables on health. When the indirect (mediated) effect is statistically significant, the bottom part of each block reports the contribution to the indirect effect of each mediator separately.

We start commenting the *total effect* of early-life conditions, i.e. their effect on health at later life not adjusting for the mediators (education and life-course trajectories). The positive and statistically significant total effects displayed in Tables 1 and 2 indicate that both for women and men early-life conditions are significantly associated with all three health outcomes. More specifically, poor health conditions during childhood and lower SES at 10 are associated with worse health in later life. In most of the cases, the associations between early-life conditions and health outcomes remain, although weakened, after adjusting for education and life-course trajectories (direct effects).

The change between the total (unadjusted) and direct (adjusted) effects of early-life conditions is often substantive and statistically significant. This means that a substantial part of the early-life influences on health are captured by educational attainment and life-course trajectories (indirect effect). This never occurs for health conditions during childhood, whose effect on health in later life does not seem to be significantly mediated by education nor by life-course histories. On the contrary, the effect of SES during childhood on older people’s health seems to be considerably captured by the considered mediators. The ratio of the indirect effect over the total effect ranges from 22% to 69% for women and from 31% to 58% (S2 Table). For both sexes, it is the highest for GALI. For example, 58% of the gap in the probability of reporting a limitation (GALI) between men with low versus high SES during childhood is explained by their educational attainment and life-course trajectories. The bottom parts of Tables 1 and 2 show what portion of the indirect effect can be attributed to each mediator. These findings indicate that each cluster of life-course trajectories explain only a marginal portion of the mediated effect (never bigger than 4.7%). Also taken together, the family and employment trajectories do not seem to mediate the effect of early-life conditions. Attaining a high level of education is what explains the biggest share of the indirect effect of early-life conditions on health (always bigger than 65%).

Next we assess relationships depicted in Fig 2, i.e., we examine the effect of early-life conditions on life-course trajectories and to what extent this effect is mediated by educational attainment. Tables 3 and 4 show the results of the KHB method applied using multinomial regressions. The reference category of the outcome variable is the most frequent cluster, i.e. *“Two children*, *married”* for both women and men. The proportions of direct and indirect effects obtained from these estimates are reported in S3 and S4 Tables. Early-life conditions are notably associated with family and employment trajectories, especially for women, as indicated by the total effects in Tables 3 and 4. For example, both women and men who reported poor health during childhood are more likely to belong to the cluster *“No children*, *no union”* than to the most frequent one (*“Two children*, *married”*).

**Table 3:** Estimated multinomial regression coefficients (standard errors in parentheses) of SES and Health at 10 (early-life conditions) on clusters of life course trajectories and mediating effect of education. Women. (N = 6,221).

Early-life conditions		Clusters of life course trajectories										
		Inactive, no union	Inactive, children 3+	Married, children 3+	Married, one child	No union, children	Emplyed, married, no children	Inactive, one child	Inactive, two children	Part-time, children 3+	Inactive, married, no children	Part-time, two children
		(n = 120)	(n = 1286)	(n = 768)	(n = 627)	(n = 292)	(n = 222)	(n = 301)	(n = 946)	(n = 304)	(n = 153)	(n = 343)
SES Medium-High	Total effect	0.21	0.15	0.11	0.05	-0.35*	0.01	0.19	0.36*	0.22	0.04	0.25
		(0.29)	(0.14)	(0.14)	(0.14)	(0.18)	(0.19)	(0.20)	(0.14)	(0.17)	(0.28)	(0.16)
	Direct effect	-0.05	-0.15	0.05	-0.02	-0.34†	-0.05	-0.09	0.07	0.07	-0.28	0.11
		(0.30)	(0.14)	(0.14)	(0.14)	(0.18)	(0.20)	(0.21)	(0.14)	(0.18)	(0.29)	(0.17)
	Indirect effect	0.26†	0.29†	0.07	0.07	-0.02	0.07	0.27†	0.29†	0.14	0.33†	0.14†
		(0.15)	(0.15)	(0.07)	(0.05)	(0.04)	(0.06)	(0.15)	(0.15)	(0.09)	(0.18)	(0.08)
SES Medium-Low	Total effect	0.58*	0.48**	0.25†	0.04	-0.61**	-0.44*	0.15	0.55***	-0.04	0.52†	0.04
		(0.28)	(0.14)	(0.14)	(0.14)	(0.20)	(0.22)	(0.21)	(0.14)	(0.20)	(0.27)	(0.18)
	Direct effect	0.23	0.07	0.14	-0.06	-0.59**	-0.54*	-0.23	0.14	-0.25	0.06	-0.15
		(0.29)	(0.14)	(0.15)	(0.15)	(0.20)	(0.23)	(0.21)	(0.15)	(0.20)	(0.28)	(0.19)
	Indirect effect	0.35*	0.41**	0.11	0.09	-0.02	0.10	0.38*	0.41**	0.21*	0.46*	0.19*
		(0.17)	(0.15)	(0.08)	(0.06)	(0.06)	(0.07)	(0.15)	(0.16)	(0.10)	(0.20)	(0.09)
SES Low	Total effect	-0.16	0.72***	0.59***	0.27†	-0.42†	-0.26	0.30	0.42**	0.55*	0.77**	0.30
		(0.36)	(0.15)	(0.15)	(0.16)	(0.21)	(0.25)	(0.22)	(0.15)	(0.21)	(0.28)	(0.22)
	Direct effect	-0.55	0.27†	0.46***	0.17	-0.39†	-0.36	-0.12	-0.03	0.30	0.25	0.09
		(0.37)	(0.15)	(0.16)	(0.17)	(0.22)	(0.26)	(0.22)	(0.16)	(0.22)	(0.29)	(0.23)
	Indirect effect	0.38*	0.45**	0.14†	0.10	-0.03	0.11	0.42**	0.46**	0.24*	0.52*	0.21*
		(0.17)	(0.16)	(0.08)	(0.06)	(0.06)	(0.08)	(0.16)	(0.16)	(0.10)	(0.20)	(0.09)
Poor Health at 10	Total effect	0.79**	-0.21	-0.12	0.03	0.45*	0.73**	-0.08	-0.10	-0.07	0.35	-0.10
		(0.28)	(0.17)	(0.18)	(0.18)	(0.21)	(0.22)	(0.26)	(0.18)	(0.23)	(0.29)	(0.23)
	Direct effect	0.76**	-0.24	-0.13	0.02	0.45*	0.72**	-0.11	-0.14	-0.08	0.31	-0.11
		(0.28)	(0.17)	(0.18)	(0.18)	(0.21)	(0.22)	(0.26)	(0.18)	(0.23)	(0.29)	(0.23)
	Indirect effect	0.03	0.03	0.01	0.01	0.00	0.01	0.03	0.03	0.02	0.04	0.02
		(0.13)	(0.15)	(0.07)	(0.04)	(0.01)	(0.04)	(0.14)	(0.15)	(0.08)	(0.17)	(0.07)

Note: All models control for age and country. Reference category of dependent variable is the cluster: Married, two children. Reference categories for SES and Health are “High SES” and “Good Health”, respectively.

*** p<0.001;

** p<0.01;

* p<0.05;

^†^ p<0.1.

Inactive refers to the labour market only.

**Table 4:** Estimated multinomial regression coefficients (standard errors in parentheses) of SES and Health at 10 (early-life conditions) on clusters of life course trajectories and mediating effect of education. Men. (N = 5,813).

Early-life conditions		Clusters of life course trajectories				
		Children 3+	No union, children	Married, no children	One child	Low employment
		(n = 1790)	(n = 273)	(n = 616)	(n = 822)	(n = 133)
SES Medium-High	Total effect	-0.06	-0.05	0.09	-0.16	-0.25
		(0.09)	(0.19)	(0.13)	(0.12)	(0.31)
	Direct effect	-0.05	-0.12	0.11	-0.14	-0.40
		(0.10)	(0.19)	(0.13)	(0.12)	(0.32)
	Indirect effect	-0.01	0.06	-0.02	-0.02	0.14
		(0.06)	(0.09)	(0.03)	(0.03)	(0.12)
SES Medium-Low	Total effect	0.08	0.09	0.00	-0.11	0.09
		(0.08)	(0.09)	(0.09)	(0.11)	(0.09)
	Direct effect	0.08	-0.01	0.03	-0.08	-0.12
		(0.08)	(-0.01)	(0.03)	(0.08)	(0.12)
	Indirect effect	0.00	0.11	-0.03	-0.03	0.21
		(0.01)	(0.11)	(0.03)	(0.03)	(0.21)
SESLow	Total effect	0.25*	0.35†	0.01	-0.09	0.08
		(0.11)	(0.20)	(0.15)	(0.13)	(0.30)
	Direct effect	0.24*	0.21	0.04	-0.06	-0.19
		(0.10)	(0.20)	(0.15)	(0.13)	(0.31)
	Indirect effect	0.01	0.15	-0.03	-0.03	0.26†
		(0.11)	(0.21)	(0.15)	(0.14)	(0.31)
Poor Health at 10	Total effect	0.13	0.54*	0.56**	0.25	0.20
		(0.06)	(0.10)	(0.05)	(0.04)	(0.14)
	Direct effect	0.13	0.53*	0.56**	0.25	0.20
		(0.13)	(0.54)	(0.56)	(0.25)	(0.2)
	Indirect effect	0.01	0.01	0.00	0.00	0.01
		(0.13)	(0.53)	(0.56)	(0.25)	(0.20)

Note: All models control for age and country. Reference category of dependent variable is the cluster: “Two children, married”. Reference categories for SES and Health are “High SES” and “Good Health”, respectively.

*** p<0.001;

** p<0.01;

* p<0.05;

^†^ p<0.1.

SES during childhood is also associated in a complex manner with the clusters of family and employment trajectories. Generally, lower SES is associated with clusters that deviate from the most common life-course trajectories. For example, women exposed to a low SES during childhood are more likely, as compared to their counterpart with a high SES background, to belong to clusters characterized by low prevalence of employment and either childlessness or fertility levels above the average (e.g., *“No children*, *married*, *inactive”*, *“Three children*, *inactive”*, *“Three children*, *married”*). However, there are also clusters for which the association with SES is reversed. For example, for women low SES is associated with a lower probability, than those with high SES, of belonging to the cluster *“No children*, *no union”*. In general, the associations between SES and clusters of life-course trajectories are more evident when considering the differences between the extreme levels (i.e., low vs high SES), whereas virtually no statistically significant difference is found between medium-high and high SES.

For men, SES during childhood is almost never significantly associated with life-course trajectories. The only exceptions are for those coming from the most disadvantaged background that, compared to those with high SES, display higher probabilities to belong to the clusters that diverge from the most common one in terms of fertility, in both directions: *“Children 3+”* and *“No children*, *no union”*.

As for the indirect effects of early-life conditions on life-course trajectories, i.e. the mediating effect of education, Table 4 indicates that they are very small and never statistically significant for men. In contrast, Table 3, shows that the effect of early-life conditions often changes dramatically when adjusting for educational attainment, pointing to an important mediating role of education for women. To cite an instance, 63% of the effect of low SES on the probability of belonging to the cluster *“Children 3+*, *inactive”* is mediated by educational attainment (S3 Table). Finally, we notice that also for women the effect of health during childhood is not mediated by education.

## Discussion and conclusions

Using data from the first three waves of the Survey of Health, Ageing and Retirement in Europe (SHARE), we examined to what extent the effects of SES and health during childhood on health in later life were mediated by educational attainment and life-course trajectories. We used multichannel sequence analysis to account for the interrelationships between three life-course dimensions (fertility, partnerships, employment). Cluster analysis was employed to identify groups of individuals who experienced similar trajectories in the three life-course dimensions. Finally, the KHB method has been used to disentangle direct and indirect effects of early-life conditions.

For both sexes, early-life conditions were found to be associated with health in later life even after adjusting for educational attainment and life-course trajectories. However, these factors explained up to 69% of the effect of childhood SES. When decomposing the contribution of each mediator, we found that attaining high education explained the largest part of the indirect effect of early-life conditions, while the mediating role of life-course trajectories was marginal. The effect of childhood health was mediated neither by education nor by life-course trajectories.

Our results confirm the adequacy of considering different models of early-life influences as complementary. More specifically, our results suggest that the Critical Period Model is more suitable to understand the effects of early-life conditions that are not mediated by other factors which is the case for childhood health, whereas the Pathway Model allows for a better understanding of the influence of socioeconomic early-life conditions on later health that is indirectly explained by mediators like educational attainment or life-course trajectories.

The second part of our analyses, aimed at deepening our understanding of the mediating role of education, in the relationship of early-life conditions and life-course trajectories. Early-life conditions were found to influence family and employment trajectories, especially for women. Generally, individuals who reported poor health or low SES during childhood were more likely to have experienced life-course that deviate from the most common patterns. For example, both women and men who reported poor childhood health were more likely to belong to the cluster characterized by childlessness and life-long singlehood. Women exposed to a low SES during childhood were more likely, to belong to clusters characterized by low prevalence of employment and either childlessness or fertility levels above the average level.

Major differences by gender were found with respect to the indirect effects of early-life conditions on life-course trajectories through education. For men the mediating effect of education was rather marginal. This was found also for women but limited to childhood health. For SES, on the contrary, we found an important mediating role of education. According to the theory of resource substitution which states that education benefits health most among people with fewer alternative resources [47], these gender differences can be explained based on fewer socioeconomic resources of women and restricted opportunities within the labor market. This analysis clarified that the finding of an insignificant mediating role of life course trajectories on health does not mean that early-life conditions do not influence family and life course trajectories; it means that this effect is almost entirely captured by educational attainment, at least for women.

This study has also some limitations. First, our sample is selected because we can only observe individuals who survived till age 60 and more. This probably implies underestimating the effect of early-life conditions because the frailest individuals who suffered from very poor health and SES during childhood may have died before. Second, although we accounted for important mediators of early-life influences, other variables or more specific characteristics of the considered dimensions may explain their remaining (direct) effects on health. For example, future studies may examine the role of the type of occupation. Third, more sophisticated analyses could be implemented. For example, future studies may examine possible interactions between early-life conditions and/or education and life course trajectories.

Despite these limitations, this study contributes to our understanding of the relationship of SES and health during childhood and health later in life. Our finding have highlighted that the effect of early-life conditions is pervasive and it branches into different mechanisms operating over the life-course, including influences on family and employment trajectories, but especially education. Education plays a double mediating role: it mediates the effect of early-life conditions on health in later life; and it mediates the effect of early-life conditions on family and employment trajectories, that in turn have been shown to be associated with health.

All in all our findings, consistently with other studies, suggest that childhood SES and health can have lasting direct effects on adult health. This indicates that public investments in children are expected to produce long lasting effects on people’s lives. Moreover, the fact that a great part of their influence passes through education means that interventions in the education system may alleviate the harmful consequences of negative experiences during childhood.

## Supporting information

S1 Table Descriptive statistics of clusters’ characteristics. (DOCX)

S2 Table Percentage of direct and indirect effects corresponding to estimates in Tables 1 and 2. (DOCX)

S3 Table Percentage of direct and indirect corresponding to estimates in Table 3. (DOCX)

S4 Table Percentage of direct and indirect corresponding to estimates in Table 4. (DOCX)

S1 Fig State distribution plots for each cluster and separately by dimension. **Women**. Note: Each graph plots the proportion of individuals in each of the different states at each time point.
(TIFF)

S2 Fig State distribution plots for each cluster and separately by dimension. **Men**. Note: Each graph plots the proportion of individuals in each of the different states at each time point.
(TIFF)
