# Supplementary material for: Early-life conditions and health at older ages: The mediating role of educational attainment, family and employment trajectories
Source: PLoS One. 2018 Apr 5;13(4):e0195320. doi: 10.1371/journal.pone.0195320 (PMC5886483; doi:10.1371/journal.pone.0195320)
Supplement: S1 Table — (DOCX) [file pone.0195320.s001.docx]

**S1 Table. Descriptive statistics of clusters' characteristics.**

| Clusters |  |  | Dimension 1: Fertility trajectories | | | |  | Dimension 2: Marital trajectories | | | | |  | Dimension 3: Employment trajectories | | | | | | |
| --- | --- | --- | --- | --- | --- | --- | --- | --- | --- | --- | --- | --- | --- | --- | --- | --- | --- | --- | --- | --- |
|  |  |  | 1st child | Total number of children (age 59) | Children out of wedlock | Childlessness |  | 1st time living with partner | | Experienced a union dissolution | Experienced  a cohabitation | Re-partnered | | Age stopped education | First age working (from 15) | | Years working (from 15 to 59) | Experienced unemployment (> 6 months consecutive) | Retired at 59 | Retired or other inactive at 59 |
|  |  | N | Mean age | Mean | % | % |  | Mean age | Never (%) | % | % | % |  | Mean | Mean age | Never (%) | Mean | % | % | % |
| *Women* |  |  |  |  |  |  |  |  |  |  |  |  |  |  |  |  |  |  |  |  |
| No union,  inactive | | 120 | 25.6 | 1.7 | 60.8 | 39.2 |  | 50.8 | 88.3 | 3.3 | 0.0 | 0.8 |  | 16.0 | 18.9 | 43.3 | 10.9 | 5.0 | 7.5 | 83.4 |
| Children 3+,  inactive | | 1,026 | 24.3 | 3.8 | 3.6 | 0.0 |  | 23.1 | 0.0 | 0.3 | 0.0 | 0.5 |  | 15.6 | 18.4 | 44.9 | 4.5 | 1.9 | 2.5 | 98.1 |
| Children 3+,  married | | 768 | 23.4 | 3.7 | 6.4 | 0.0 |  | 22.4 | 0.0 | 0.4 | 0.0 | 1.0 |  | 16.7 | 20.5 | 0.0 | 31.5 | 8.3 | 37.2 | 53.2 |
| Two children,  married | | 1,119 | 24.8 | 2.0 | 3.1 | 0.0 |  | 23.0 | 0.0 | 1.1 | 0.0 | 0.6 |  | 17.3 | 19.9 | 0.0 | 33.7 | 10.3 | 44.1 | 54.0 |
| One child,  married | | 627 | 27.6 | 1.0 | 3.0 | 0.0 |  | 24.5 | 0.0 | 1.0 | 0.2 | 1.0 |  | 17.2 | 19.2 | 0.0 | 34.0 | 12.6 | 33.2 | 53.3 |
| Children,  no union | | 292 | 27.4 | 0.2 | 15.1 | 84.9 |  | 41.6 | 87.3 | 1.4 | 9.6 | 0.0 |  | 17.5 | 20.1 | 0.0 | 36.1 | 13.7 | 27.7 | 42.5 |
| No children,  married, employed | | 222 | -- | 0.0 | 0.0 | 100.0 |  | 29.4 | 0.0 | 0.9 | 1.4 | 0.9 |  | 17.4 | 19.2 | 0.0 | 35.2 | 10.4 | 25.2 | 51.8 |
| One child,  inactive | | 301 | 27.7 | 1.0 | 2.7 | 0.0 |  | 24.6 | 0.0 | 0.0 | 0.0 | 0.3 |  | 15.8 | 18.6 | 45.2 | 5.9 | 3.3 | 3.7 | 97.5 |
| Two children,  inactive | | 946 | 25.6 | 2.0 | 2.0 | 0.0 |  | 23.9 | 0.0 | 0.3 | 0.0 | 0.1 |  | 15.8 | 18.1 | 45.5 | 6.2 | 1.8 | 3.2 | 98.0 |
| Children 3+,  part-time | | 304 | 23.9 | 3.4 | 4.3 | 0.0 |  | 22.7 | 0.0 | 1.0 | 0.0 | 0.0 |  | 17.2 | 19.6 | 0.0 | 29.3 | 8.6 | 9.9 | 29.9 |
| No children,  married, inactive | | 153 | 31.0 | 0.0 | 0.0 | 99.3 |  | 25.0 | 0.0 | 0.7 | 0.0 | 0.0 |  | 15.6 | 19.1 | 41.8 | 7.6 | 3.9 | 6.5 | 93.5 |
| Two children,  part-time | | 343 | 25.3 | 2.0 | 3.2 | 0.0 |  | 23.0 | 0.0 | 1.2 | 0.0 | 0.6 |  | 17.4 | 18.9 | 0.0 | 32.8 | 7.9 | 16.6 | 29.4 |
| Total |  | 6,221 | 25.1 | 2.2 | 4.9 | 10.7 |  | 23.8 | 5.8 | 0.7 | 0.5 | 0.5 |  | 16.6 | 19.4 | 18.4 | 21.9 | 7.0 | 20.9 | 68.2 |
| *F-test* |  |  | 55.3* | 1391.5* |  |  |  | 149.9* |  |  |  |  |  | 55.6* | 7.8* |  | 1596.3* |  |  |  |
| *Kruskal-Wallis* |  |  | 419.9* | 5293.2* |  |  |  | 342.9* |  |  |  |  |  | 618.8* | 157.9* |  | 4190.1* |  |  |  |
| *Pearson Chi^2^* |  |  |  |  | 922.7* | 5500* |  |  | 5400* | 25.0* | 500.7* | 14.4 |  |  |  | 2000* |  | 167.7* | 1800* | 6100* |
| *Men* |  |  |  |  |  |  |  |  |  |  |  |  |  |  |  |  |  |  |  |  |
| Children 3+ |  | 1,790 | 27.1 | 3.6 | 4.7 | 0.0 |  | 25.8 | 0.0 | 0.6 | 0.0 | 1.2 |  | 17.3 | 18.8 | 0.0 | 38.8 | 7.6 | 23.2 | 34.2 |
| Children, no union | | 273 | 48.7 | 0.0 | 0.4 | 98.9 |  | 47.5 | 81.3 | 0.7 | 11.0 | 0.4 |  | 17.2 | 19.2 | 6.2 | 35.0 | 13.6 | 19.4 | 37.0 |
|  | |  |  |  |  |  |  |  |  |  |  |  |  |  |  |  |  |  |  |  |
| No children, married | | 616 | 36.9 | 0.9 | 8.4 | 55.8 |  | 31.9 | 6.2 | 1.3 | 1.1 | 1.3 |  | 17.4 | 18.9 | 0.0 | 38.3 | 12.5 | 21.3 | 32.3 |
| One child |  | 822 | 30.0 | 1.0 | 3.2 | 0.0 |  | 26.9 | 0.0 | 1.0 | 0.0 | 0.9 |  | 17.5 | 18.8 | 0.0 | 38.5 | 10.7 | 28.0 | 38.1 |
| Two children | | 2,179 | 27.4 | 2.0 | 2.1 | 0.0 |  | 25.5 | 0.0 | 0.7 | 0.0 | 0.8 |  | 17.7 | 19.1 | 0.1 | 38.6 | 8.4 | 24.6 | 35.1 |
| Low employment | | 133 | 29.0 | 2.4 | 3.0 | 7.6 |  | 27.1 | 0.0 | 0.8 | 0.0 | 0.8 |  | 16.0 | 19.7 | 48.1 | 10.4 | 5.3 | 9.8 | 86.5 |
| Total |  | 5,813 | 28.3 | 2.1 | 3.7 | 10.8 |  | 26.7 | 4.5 | 0.8 | 0.6 | 1.0 |  | 17.5 | 18.9 | 1.4 | 37.8 | 9.1 | 23.7 | 36.2 |
| *F-test* |  |  | 311.6* | 2681.6* |  |  |  | 471.8* |  |  |  |  |  | 9.3* | 1.6 |  | 545.7* |  |  |  |
| *Kruskal-Wallis* |  |  | 674.9* | 4606.5* |  |  |  | 601.3* |  |  |  |  |  | 95.4* | 14.4* |  | 291.1* |  |  |  |
| *Pearson Chi^2^* |  |  |  |  | 70.4* | 4100* |  |  | 4000* | 3.7 | 496.5* | 3.5 |  |  |  | 2200* |  | 26.5* | 155.1* | 669.1* |

Note: * p < 0.05
