# Supplementary material for: Early-life conditions and health at older ages: The mediating role of educational attainment, family and employment trajectories
Source: PLoS One. 2018 Apr 5;13(4):e0195320. doi: 10.1371/journal.pone.0195320 (PMC5886483; doi:10.1371/journal.pone.0195320)
Supplement: S2 Table — (DOCX) [file pone.0195320.s002.docx]

**S2 Table. Percentage of direct and indirect effects corresponding to estimates in Tables 1 and 2.**

|  | Outcomes: | Self-perceived health | | | | | | | | Depression | | | | | | | | GALI | | | | | | | |
| --- | --- | --- | --- | --- | --- | --- | --- | --- | --- | --- | --- | --- | --- | --- | --- | --- | --- | --- | --- | --- | --- | --- | --- | --- | --- |
|  | Early life conditions: | Medium-high SES | | Medium-low SES | | Low SES | | Health | | Medium-high SES | | Medium-low SES | | Low SES | | Health | | Medium-high SES | | Medium-low SES | | Low SES | | Health | |
|  |  |  |  |  |  |  |  |  |  |  |  |  |  |  |  |  |  |  |  |  |  |  |  |  |  |
| Women (Table 1) | Direct effect | 58.1% | † | 70.1% | *** | 74.4% | *** | 98.9% | *** | 4.4% |  | 61.4% | * | 77.7% | *** | 97.9% | *** | 27.2% |  | 31.4% |  | 58.9% | ** | 98.6% | *** |
|  | Indirect effect | 41.9% |  | 29.9% | * | 25.6% | * | 1.1% |  | 95.6% |  | 38.6% | * | 22.3% | * | 2.1% |  | 72.8% | † | 68.6% | * | 41.1% | ** | 1.4% |  |
| Men (Table 2) | Direct effect | 63.3% | † | 68.8% | *** | 64.0% | *** | 98.6% | *** | 58.3% |  | 63.6% | * | 65.5% | *** | 98.0% | *** | 33.3% |  | 47.8% |  | 42.9% | ** | 100.0% | *** |
|  | Indirect effect | 36.7% |  | 31.3% | * | 36.0% | * | 1.4% |  | 41.7% |  | 36.4% | * | 34.5% | * | 2.0% |  | 66.7% | † | 52.2% | * | 57.1% | ** | 0.0% |  |

Note: the percentage of the direct and the indirect effect are calculated on the sum of the two coefficients taken in absolute value. *** p<0.001; ** p<0.01; * p<0.05; † p<0.1.
