# Supplementary material for: Early-life conditions and health at older ages: The mediating role of educational attainment, family and employment trajectories
Source: PLoS One. 2018 Apr 5;13(4):e0195320. doi: 10.1371/journal.pone.0195320 (PMC5886483; doi:10.1371/journal.pone.0195320)
Supplement: S3 Table — (DOCX) [file pone.0195320.s003.docx]

**S3 Table. Percentage of direct and indirect effects corresponding to estimates in Table 3.**

| Early-life conditions | | Clusters of life course trajectories | | | | | | | | | | | | | | | | | | | | | |
| --- | --- | --- | --- | --- | --- | --- | --- | --- | --- | --- | --- | --- | --- | --- | --- | --- | --- | --- | --- | --- | --- | --- | --- |
|  |  | Inactive, no union | | Inactive, children 3+ | | Married, children 3+ | | Married, one child | | No union, children | | Emplyed, married, no children | | Inactive, one child | | Inactive, two children | | Part-time, children 3+ | | Inactive, married, no children | | Part-time, two children | |
| SES Medium-High | Direct effect | 16.1% |  | 34.1% |  | 41.7% |  | 22.2% |  | 94.4% | † | 41.7% |  | 25.0% |  | 19.4% |  | 33.3% |  | 45.9% |  | 44.0% |  |
|  | Indirect effect | 83.9% | † | 65.9% | † | 58.3% |  | 77.8% |  | 5.6% |  | 58.3% |  | 75.0% | † | 80.6% | † | 66.7% |  | 54.1% | † | 56.0% | † |
| SES Medium-Low | Direct effect | 39.7% |  | 14.6% |  | 56.0% |  | 40.0% |  | 96.7% | ** | 84.4% | * | 37.7% |  | 25.5% |  | 54.3% |  | 11.5% |  | 44.1% |  |
|  | Indirect effect | 60.3% | * | 85.4% | ** | 44.0% |  | 60.0% |  | 3.3% |  | 15.6% |  | 62.3% | * | 74.5% | ** | 45.7% | * | 88.5% | * | 55.9% | * |
| SES Low | Direct effect | 59.1% |  | 37.5% | † | 76.7% | *** | 63.0% |  | 92.9% | † | 76.6% |  | 22.2% |  | 6.1% |  | 55.6% |  | 32.5% |  | 30.0% |  |
|  | Indirect effect | 40.9% | * | 62.5% | ** | 23.3% | † | 37.0% |  | 7.1% |  | 23.4% |  | 77.8% | ** | 93.9% | ** | 44.4% | * | 67.5% | * | 70.0% | * |
| Health at 10 | Direct effect | 96.2% | ** | 88.9% |  | 92.9% |  | 66.7% |  | 100.0% | * | 98.6% | ** | 78.6% |  | 82.4% |  | 80.0% |  | 88.6% |  | 84.6% |  |
|  | Indirect effect | 3.8% |  | 11.1% |  | 7.1% |  | 33.3% |  | 0.0% |  | 1.4% |  | 21.4% |  | 17.6% |  | 20.0% |  | 11.4% |  | 15.4% |  |

Note: the percentage of the direct and the indirect effect are calculated on the sum of the two coefficients taken in absolute value. *** p<0.001; ** p<0.01; * p<0.05; † p<0.1.
