# Supplementary material for: Early-life conditions and health at older ages: The mediating role of educational attainment, family and employment trajectories
Source: PLoS One. 2018 Apr 5;13(4):e0195320. doi: 10.1371/journal.pone.0195320 (PMC5886483; doi:10.1371/journal.pone.0195320)
Supplement: S4 Table — (DOCX) [file pone.0195320.s004.docx]

**S4 Table. Percentage of direct and indirect effects corresponding to estimates in Table 4.**

| Early-life conditions | | Clusters of life course trajectories | | | | | | | | | |
| --- | --- | --- | --- | --- | --- | --- | --- | --- | --- | --- | --- |
|  |  | Children 3+ | | No union, children | | Married, | | One child | | Low employment | |
|  |  |  |  |  |  | no children | |  |  |  |  |
|  |  | (n=1790) | | (n=273) | | (n=616) | | (n=822) | | (n=133) | |
| SES Medium-High | Direct effect | 83.3% |  | 66.7% |  | 84.6% |  | 87.5% |  | 74.1% |  |
|  | Indirect effect | 16.7% |  | 33.3% |  | 15.4% |  | 12.5% |  | 25.9% |  |
| SES Medium-Low | Direct effect | 100.0% |  | 8.3% |  | 50.0% |  | 72.7% |  | 36.4% |  |
|  | Indirect effect | 0.0% |  | 91.7% |  | 50.0% |  | 27.3% |  | 63.6% |  |
| SESLow | Direct effect | 96.0% | * | 58.3% |  | 57.1% |  | 66.7% |  | 42.2% |  |
|  | Indirect effect | 4.0% |  | 41.7% |  | 42.9% |  | 33.3% |  | 57.8% | † |
| Health child | Direct effect | 92.9% |  | 98.1% | * | 100.0% | ** | 100.0% |  | 95.2% |  |
|  | Indirect effect | 7.1% |  | 1.9% |  | 0.0% |  | 0.0% |  | 4.8% |  |
|  |  |  |  |  |  |  |  |  |  |  |  |

Note: the percentage of the direct and the indirect effect are calculated on the sum of the two coefficients taken in absolute value.

*** p<0.001; ** p<0.01; * p<0.05; † p<0.1.
