# Supplementary figures and images for: Early-life conditions and health at older ages: The mediating role of educational attainment, family and employment trajectories
Source: PLoS One. 2018 Apr 5;13(4):e0195320. doi: 10.1371/journal.pone.0195320 (PMC5886483; doi:10.1371/journal.pone.0195320)

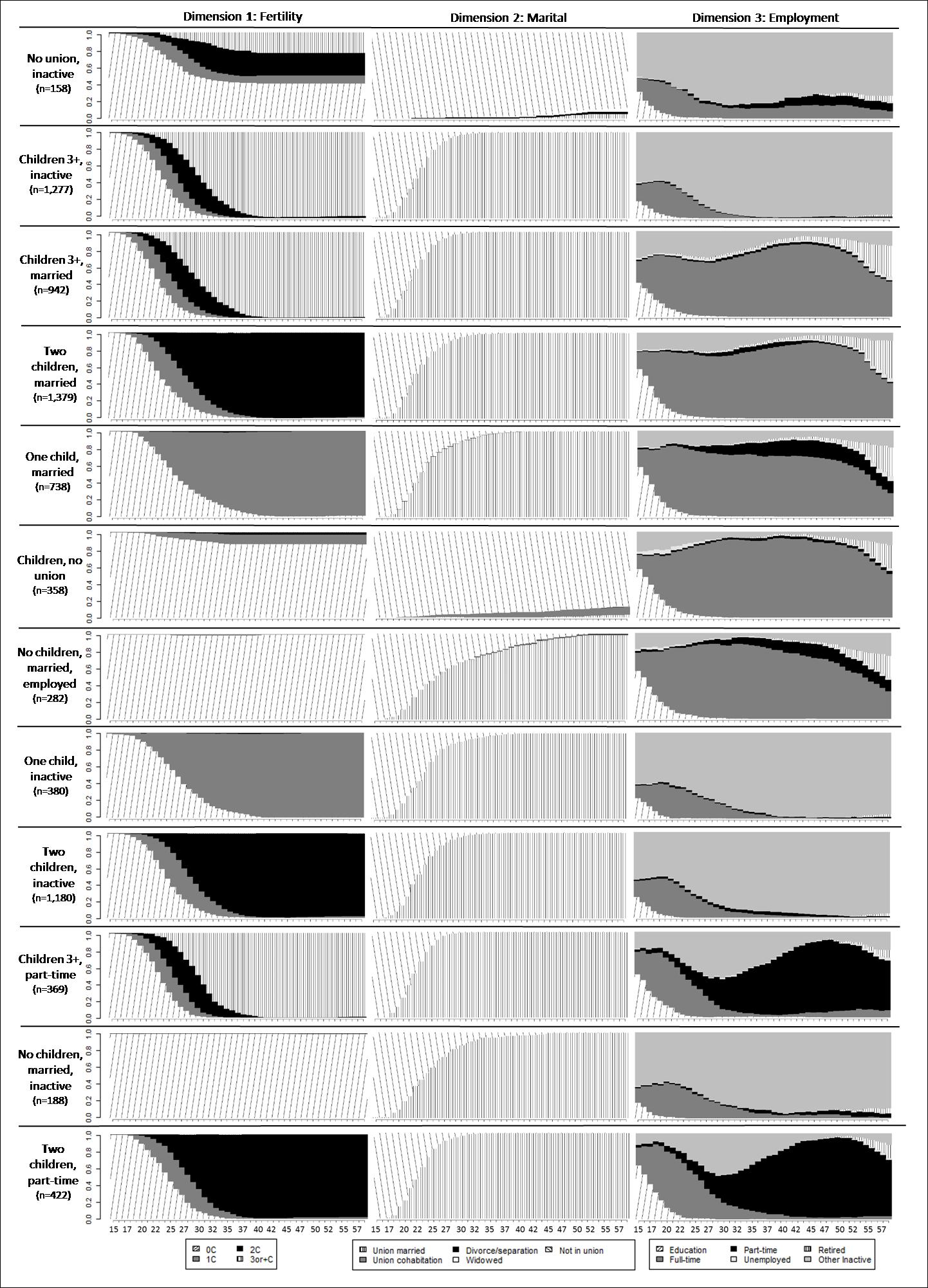

Supplement: S1 Fig — Women. Note: Each graph plots the proportion of individuals in each of the different states at each time point. (TIFF) [file pone.0195320.s005.tiff]

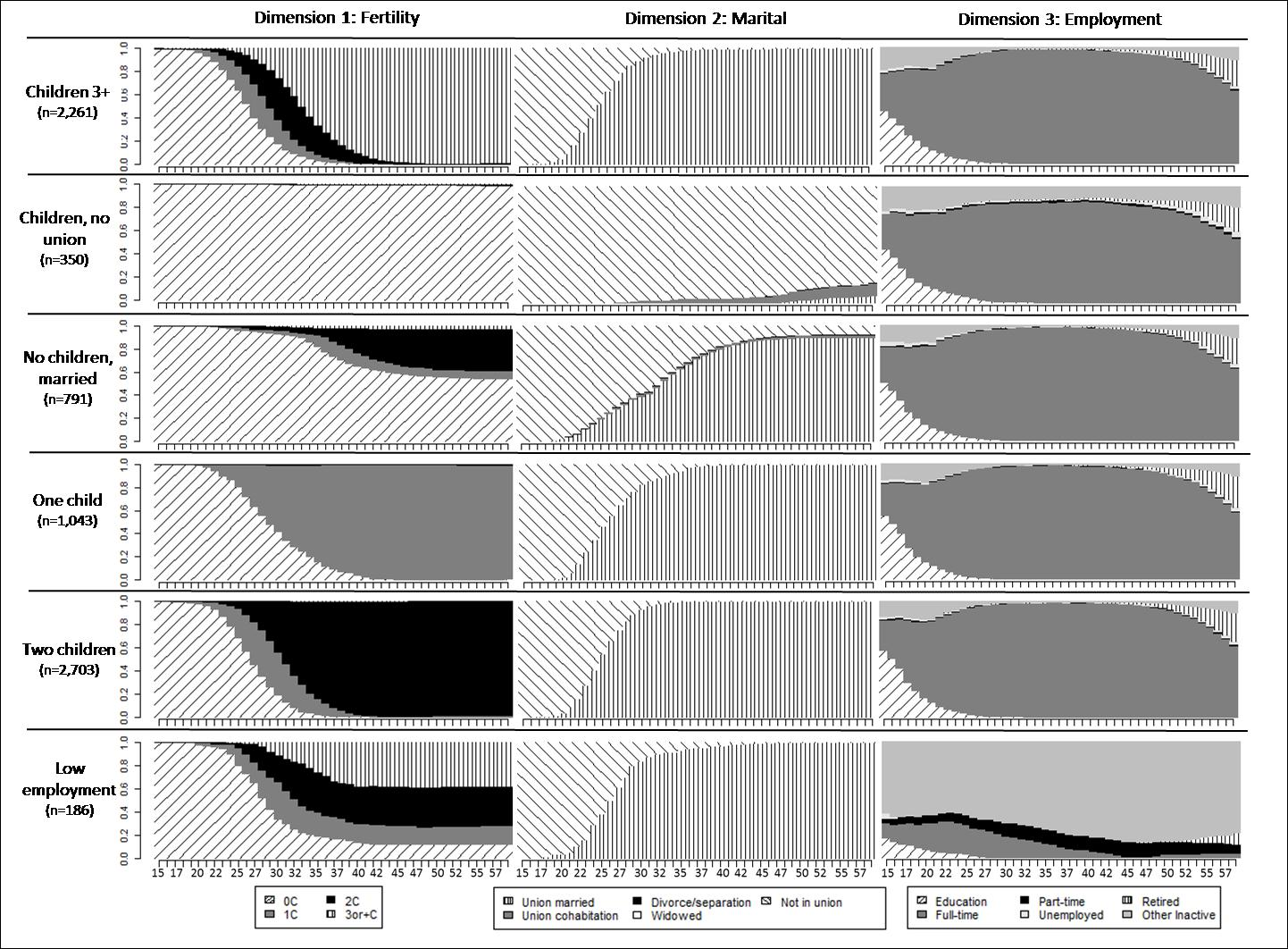

Supplement: S2 Fig — Men. Note: Each graph plots the proportion of individuals in each of the different states at each time point. (TIFF) [file pone.0195320.s006.tiff]
